# Oral treprostinil improves pulmonary vascular compliance in pulmonary arterial hypertension

**DOI:** 10.1016/j.rmed.2022.106744

**Published:** 2022-01-19

**Authors:** Akram Khan, R. James White, Gisela Meyer, Tomas R. Pulido Zamudio, Carlos Jerjes-Sanchez, Dana Johnson, Rob Grover, Meredith Broderick, Aliou Ousmanou, Louis Holdstock, Evangelos Michelakis

**Affiliations:** a Division of Pulmonary and Critical Care Medicine, School of Medicine, Oregon Health and Science University, Portland, OR, USA; b Division of Pulmonary & Critical Care Medicine and the Mary M. Parkes Center, University of Rochester Medical Center, Rochester, NY, USA; c Complexo Hospitalar Santa Casa de Porto Alegre, Porto Alegre, Brazil; d Cardiopulmonary Department, Ignacio Chávez National Heart Institute, Mexico City, Mexico; e Tecnologico de Monterrey, Escuela de Medicina y Ciencias de la Salud, Instituto de Cardiologia y Medicina Vascular, TEC Salud, San Pedro Garza Garcia, Nuevo Leon, Mexico; f Unidad De Investigación Clinica en Medicina, Monterrey, Mexico; g United Therapeutics, Research Triangle Park, NC, USA; h Department of Medicine, Alberta Cardiovascular and Stroke Research Centre, University of Alberta, Edmonton, Canada

**Keywords:** FREEDOM-EV, Right-heart catherization, PVR, Hemodynamics, Cardiac output

## Abstract

Oral treprostinil has been shown to improve exercise capacity and delay disease progression in patients with pulmonary arterial hypertension (PAH), but its effects on hemodynamics are not well-characterized. The FREEDOM-EV trial was a Phase III, international, placebo-controlled, double-blind, event-driven study in 690 participants with PAH who were taking a single oral PAH therapy. FREEDOM-EV demonstrated a significantly reduced risk for clinical worsening with oral treprostinil taken three times daily and did not uncover new safety signals in PAH patients. Sixty-one participants in the FREEDOM-EV trial volunteered for a hemodynamics sub- study. Pulmonary artery compliance (PAC), a ratio of stroke volume to pulmonary pulse pressure, significantly increased from Baseline to Week 24 in the oral treprostinil group compared with the placebo group (geometric mean 26.4% active vs. −6.0% placebo; ANCOVA p=0.007). There was a significant increase in cardiac output in the oral treprostinil group compared to the placebo group (geometric mean 11.3% active vs. −6.4% placebo; ANCOVA p=0.005) and a corresponding significant reduction in pulmonary vascular resistance (PVR) (geometric mean −21.5 active vs. −1.8% placebo; ANCOVA p=0.02) from Baseline to Week 24. These data suggest that increased compliance contributes to the physiological mechanism by which oral treprostinil improves exercise capacity and delays clinical worsening for patients with PAH.

FREEDOM-EV demonstrated a significantly reduced risk for clinical worsening in patients with pulmonary arterial hypertension (PAH) who took oral treprostinil three times daily [1]. Prospective hemodynamics studies of oral treprostinil, however, are limited [2]. A subset of participants in the FREEDOM-EV trial consented to a right heart catheterization (RHC) at Baseline and study Week 24. Some of the results were previously reported in abstract form [3].

## 1. Methods

Sixty-one participants of the FREEDOM-EV (NCT01560624) study underwent two RHCs (34 oral treprostinil, 27 placebo) [1]. All participants were on a stable dose of oral PAH monotherapy at study entry. The baseline RHC was performed prior to the first study drug dose; follow-up RHC was performed within 72 h of Week 24 assessments. When possible, RHC was performed 3–6 h after study drug administration. For this optional sub-study, investigator assessments were accepted without a core lab review; no reproducibility requirements for thermodilution or pressures were specified. Pulmonary artery compliance (capacitance) was calculated as [(cardiac output/heart rate)/(systolic pulmonary artery pressure – diastolic pulmonary artery pressure)]. Heart rate was collected once during each RHC.

Six participants with missing or mismatched CO measurement methodology, Fick (direct or indirect) or thermodilution, at baseline and follow up were not included in CO-based analyses. The treatment groups were compared using analysis of covariance with change from baseline for each log-transformed hemodynamic parameter as the dependent variable, treatment as a fixed effect, and log-transformed baseline hemodynamic parameter as a covariate.

## 2. Results

### 2.1 Demographics and baseline characteristics

The sub-study treatment groups were generally balanced (Table 1), but those treated with oral treprostinil had greater median height and weight than placebo treated participants (p = 0.005 and 0.007, respectively). Although median time since diagnosis was significantly longer in the oral treprostinil group compared to placebo (9.7 vs 2.9 months, p = 0.05), background PAH therapies and time on those therapies were similar. The 54 participants with matched cardiac output measures had similar demographics (Supplemental Table E1).

### 2.2 Hemodynamic outcomes

**Fig. 1.**
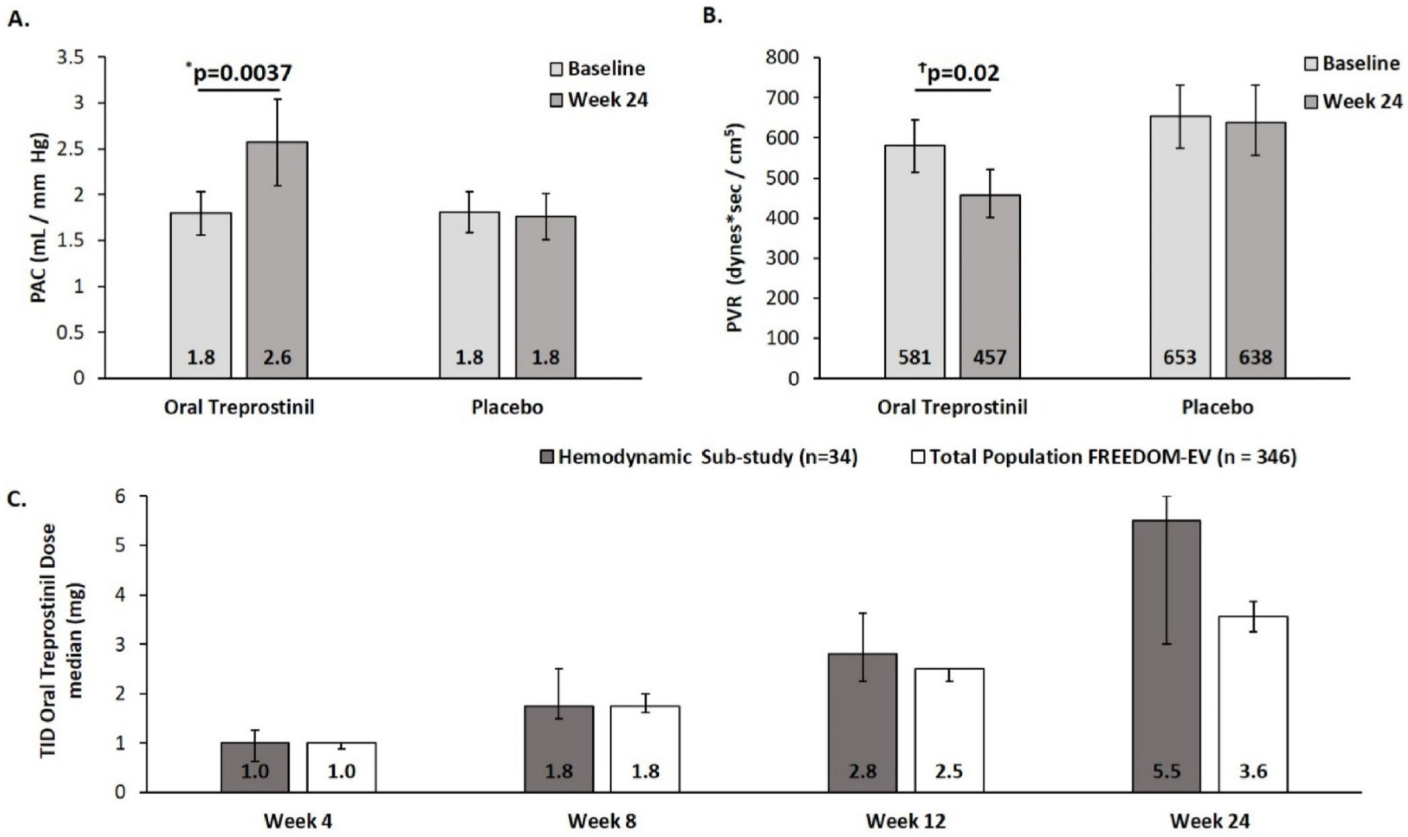
FREEDOM-EV Hemodynamic Sub-study. A) Mean (unadjusted) pulmonary arterial compliance increased by 44% from baseline to Week 24 in the oral treprostinil (n = 30) group versus 0% in the placebo (n = 24) group. Values represent means; error bars represent mean ± standard error B) Geometric mean (unadjusted) pulmonary vascular resistance dropped 21% from baseline to Week 24 in the oral treprostinil (n = 30) group versus 2% in the placebo (n = 24) group. Values represent geometric means; error bars represent geometric mean multiplied/divided by geometric standard error. C) Comparison of median dose for the hemodynamic sub-study to the total population of FREEDOM-EV over time. Error bars represent the 95% confidence interval. *p-value is obtained from the analysis of covariance with change from baseline in raw PAC as the dependent variable, treatment as fixed effect, and baseline raw PAC as a covariate. ^Ϯ^p-value is obtained from the analysis of covariance with change from baseline in log-transformed PVR as the dependent variable, treatment as fixed effect, and log-transformed baseline PVR as a covariate. PAC: pulmonary artery compliance; TID: three times a day; PVR: pulmonary vascular resistance.

Mean PAC increased 44% at Week 24 (raw mean, Fig. 1a) in treprostinil treated patients compared to stable measurements for placebo; in a model with log-transformed PAC that adjusts for baseline, the increase in geometric mean was 26% (Table 2) in treprostinil treated patients compared to a 6% decrease in the geometric mean for placebo (Table 2). CO increased for oral treprostinil participants relative to placebo (Table 2), and PVR decreased (Table 2, Fig. 1b). No significant changes were found in PAWPm, PAPm, SAPm, RAPm, SvO2, or SaO2. Analyses based on the raw data are shown in the supplement (Table E2). In patients receiving oral treprostinil, NT-proBNP was not statistically lower at Week 24 (Table 2).

Median oral treprostinil doses (Fig. 1c) at Week 24 were higher in the hemodynamic sub-study (5.5 mg TID, CI 3.0–6.0 mg) as compared to 3.6 mg TID (CI 3.3–3.9 mg) in the parent FREEDOM-EV study. The adverse event profile in sub-study participants was similar to the overall FREEDOM-EV population, despite the higher average oral treprostinil dose achieved (data not shown) [1].

## 3. Discussion

This FREEDOM-EV hemodynamic sub-study is the first prospective, placebo-controlled study to examine the effects of oral treprostinil taken three times daily in PAH patients. Treprostinil treatment resulted in a significant increase in PAC and CO with an associated reduction in PVR compared to the placebo group at Week 24. These data can be explained by the known pulmonary vasodilator effect of prostacyclin therapies [4–6]. Compliance, a ratio of stroke volume to pulmonary pulse pressure, represents the pulsatile component of afterload in PAH in contrast to the static component of resistance. In PAH, RV failure occurs with a combined increase in PVR and decrease in PAC [7]. Lankhaar et al. proposed that patients may have larger changes in compliance than resistance at earlier stages of disease [8]. In a bivariate analysis, PAC at diagnosis was the sole independent predictor of mortality (over PVR or CI) and was the most discriminating variable in receiver-operator curves [9]. Interestingly, the large improvement in compliance we observed with oral treprostinil resulted in PAC values within the lowest quartile of risk in that study. Recent registry work from the Italian network of investigators did not assess compliance but did suggest that large reductions in PVR correlate with improvements in RV function and risk scores [10,11].

Treatment groups were generally well-balanced at baseline and similar to the parent study [1]. The sub-study participants achieved a higher median dose of oral treprostinil by Week 24 (5.5 mg TID) than the parent study (3.6 mg TID). The hemodynamic improvements described here may explain the marked reductions in NT-pro-BNP observed in the entire FREEDOM-EV population [1].

Previous data on the hemodynamic effects of oral treprostinil are limited [2]. An open-label study investigated the safety and tolerability of transitioning patients from parenteral treprostinil infusion to oral treprostinil, and found no difference in CO, CI, PVR, SVR at Week 24 [2]. Three patients had late clinical worsening with increased PVR after Week 24 and returned to parenteral drug. Because the primary goal of that study was a successful transition from parenteral to oral treprostinil in a very select group of PAH patients, it is not surprising that hemodynamic parameters were largely unchanged.

Prospective hemodynamic studies of prostacyclin receptor agonists have had mixed results. In a phase II study of 61 PAH patients on mono- or combination therapy, ralinepag demonstrated significant improvements in PVR, SVR and PAPm compared to placebo at Week 22 [12]. A post-hoc analysis of the study correlated ralinepag plasma levels with ralinepag dose and with improvements in PVR and 6MWD [13]. The phase II selexipag study of 43 participants (approximately 70% on background monotherapy and 30% combination) demonstrated a reduction in PVR and increase in CI at Week 17 [14]. Conversely, in the larger TRITON study, selexipag as part of an initial triple therapy regimen did not result in hemodynamic benefit as compared to placebo (initial macitentan and tadalafil) participants who had a 50% reduction in PVR [15].

In summary, in patients with PAH, addition of oral treprostinil to approved oral monotherapy improved PAC, PVR, and CO. These improvements combined with the anti-platelet and anti-inflammatory properties of oral treprostinil may have contributed to the significant reduction in risk-adjusted clinical worsening in FREEDOM-EV [1,4–6]. These data suggest that increased compliance contributes to the physiological mechanism by which oral treprostinil reduces NT-pro-BNP and delays disease progression for patients with PAH [1,16].

## Supplementary Material

Supplementry Material

## Figures and Tables

**Table 1:** Baseline demographics for all participants in the FREEDOM-EV hemodynamic sub-study.

	Placebo (n = 27)	Oral Treprostinil (n = 34)	Overall (n = 61)	p- value^a^

Mean Age ± SD (years)	40.1 ± 14.6	44.1 ± 14.4	42.3 ± 14.5	0.30
Female/Male	23/4	23/11	46/15	0.14
Race, n (%)				0.14
White	18 (66.7)	27 (79.4)	45 (73.8)	
Asian	6 (22.2)	7 (20.6)	13 (21.3)	
Black or African American	3 (11.1)	0 (0.0)	3 (4.9)	
Median Weight (kg) (IQR)	58.0 (52.0–79.2)	75.0 (63.2–92.5)	68.0 (56.0–86.0)	**0.007**
Median Height (cm) (IQR)	159 (154–164)	164 (160–170)	162 (155–167))	**0.005**
Geographic Region n (%)				0.11
North America	5 (18.5)	12 (35.3)	17 (27.9)	
South and Latin America	17 (63.0)	12 (35.3)	29 (47.5)	
Asia-Pacific	5 (18.5)	8 (23.5)	13 (21.3)	
Europe	0 (0.0)	2 (5.9)	2 (3.3)	
Median Time Since Diagnosis (months), (IQR)	2.9 (0.1–9.6)	9.7 (3.4–39.1)	6.6 (1.9–16.1)	**0.05**
Background PAH Therapy n (%)				1.00
PDE5-I or sGC Stimulator	23 (85.2)	29 (85.3)	52 (85.2)	
ERA	4 (14.8)	5 (14.7)	9 (14.8)	
Median Time on Background PAH Therapy^b^ (months), (IQR)	6.2 (2.6–10.0)	6.5 (3.5–12.4)	6.4 (2.9–10.4)	0.67
Risk Stratification by number of low-risk criteria met, n (%)^c^				0.90
0	3 (11)	4 (12)		
1	12 (44)	11 (33)		
2	7 (26)	11 (33)		
3	5 (19)	7 (21)		

a p-values were calculated using Wilcoxon rank sum test for continuous variables and Fisher’s exact test for categorical variables.

b Imputed first day of month for participants missing day of start.

c Low-risk criteria defined as WHO functional class I or II, 6MWD greater than 440 m, and/or N-terminal pro–brain natriuretic peptide less than 300 pg/ml. Risk criteria met were only counted for subjects with all three measures available. Abbreviations: ERA, endothelin receptor antagonist; PAH, pulmonary arterial hypertension; PDE5-I, phosphodiesterase type 5 inhibitor; sGC, soluble guanylate cyclase.

**Table 2:** Summary of hemodynamic parameters.

	Placebo				Oral Treprostinil				p-value^a^
	N	Baseline	Week 24	% Change (95% CI)	n	Baseline	Week 24	% Change (95% CI)	

**PAC (mL/mmHg)**	24	1.5	1.4	− 6.0 (− 19.7–9.9)	30	1.5	1.9	26.4 (9.9–45.5)	**0.007**
PVR (dynes*sec/cm^5^)	24	653	638	− 1.8 (− 15.0–13.4)	30	581	457	− 21.5 (− 31.0–− 10.8)	**0.02**
**CO (L/min)** ^b^	24	4.5	4.3	− 6.4 (− 14.3–2.3)	30	4.9	5.4	11.3 (2.9–20.5)	**0.005**
**CI (L/min/m^2^)**	22	2.8	2.6	− 8.1 (− 16.0–0.7)	26	2.9	3.1	7.8 (− 0.8 – 17.3)	**0.01**
**RAPm (mmHg)**	24	7.0	6.7	− 1.1 (− 19.8–22.0)	33	6.8	6.6	− 4.2 (− 19.9–14.5)	0.82
**PAPm (mmHg)**	27	46.7	44.4	− 4.9 (− 13.4–4.4)	34	47.1	43.1	− 8.2 (− 15.6–_−_ 0.2)	0.57
**PAWPm (mmHg)**	26	8.1	7.9	− 8.0 (− 22.0–8.6)-	33	9.5	10.0	9.9 (− 5.1 – 27.3)	0.12
**SAPm (mmHg)** SVR (dynes*sec/cm^5^)	27 24	85.4 1366	85.4 1448	− 0.6 (− 5.6 – 4.6) 6.7 (− 4.2 – 18.7)	33 29	88.9 1313	89.1 1203	0.7 (− 3.9 – 5.5) − 8.9 (− 17.3–0.5)	0.72 **0.03**
**SvO2 (%)**	21	65.2	69.1	5.2 (0.4–10.2)	26	66.9	67.5	1.4 (− 2.7 – 5.8)	0.25
**SaO2 (%)**	26	95.7	95.4	− 0.23 (− 1.6 – 1.2)	32	94.9	94.7	− 0.4 (− 1.6 – 0.9)	0.89
**NT-proBNP (ng/mL)**	27	360	374	5.1 (− 27.0–51.3)	33	334	277	− 18.0 (− 41.0–14.0)	0.32

Hemodynamic parameters are expressed as geometric means. Only subjects with both baseline and Week 24 hemodynamic measures were included. Systemic arterial pressure measurements were noninvasive.

a p-value, % change in the geometric mean, and its associated 95% CI are obtained from the analysis of covariance with change from baseline in log-transformed data for each hemodynamic parameter as the dependent variable, treatment as fixed effect, and log-transformed baseline hemodynamic parameter as a covariate.

b Thermodilution measures were used for 17 placebo and 19 treprostinil participants, the remaining 7 and 11 participants were measured using Fick. Abbreviations: PAC, pulmonary artery compliance, CO, cardiac output; CI, cardiac index; PVR, pulmonary vascular resistance; SVR, systemic vascular resistance; PAWPm, pulmonary artery wedge pressure mean; PAPm, pulmonary artery pressure mean; SAPm, systemic arterial pressure mean; RAPm, right atrial pressure mean; SaO2, arterial oxygen saturation; SvO2, mixed venous oxygen saturation; NT-proBNP, N-terminal proB-type natriuretic peptide.

## ABBREVIATIONS

CI: cardiac index
CO: cardiac output
HR: heart rate
NT-proBNP: N-terminal proB-type natriuretic peptide
PAC: pulmonary artery compliance
PAPd: pulmonary artery pressure diastolic
PAPm: pulmonary artery pressure mean
PAPs: pulmonary artery pressure systolic
PAWPm: pulmonary artery wedge pressure mean
PVR: Pulmonary vascular resistance
RAPm: right atrial pressure mean
RV: right ventricle
SAPd: systemic arterial pressure diastolic
SAPm: systemic arterial pressure mean
SAPs: systemic arterial pressure systolic
SaO2: arterial oxygen saturation
SvO2: mixed venous oxygen saturation
SVR: systemic vascular resistance
