# Supplementary material for: Oral treprostinil improves pulmonary vascular compliance in pulmonary arterial hypertension
Source: Respir Med. Author manuscript; Available in PMC 2023 Mar 18. (PMC10024312; doi:10.1016/j.rmed.2022.106744)
Supplement: Supplementry Material [file NIHMS1849918-supplement-Supplementry_Material.docx]

**Supplemental Data**

**Supplemental Table E1. Baseline Demographics for Participants with Matched Cardiac Output Methodology in the FREEDOM-EV Hemodynamic Sub-study**

|  | Placebo  (n=24) | Oral Treprostinil (n=30) | Overall  (n=54) | p-value* |
| --- | --- | --- | --- | --- |
| Mean Age ± SD (years) | 39.9 (15.2) | 45.2 (14.6) | 42.8 (15.0) | 0.19 |
| Female/Male | 20/4 | 22/8 | 42/12 | 0.51 |
| Race, n (%) |  |  |  | 0.12 |
| White | 16 (66.7) | 25 (83.3) | 41 (75.9) |  |
| Asian | 5 (20.8) | 5 (16.7) | 10 (18.5) |  |
| Black or African American | 3 (12.5) | 0 (0) | 3 (5.6) |  |
| Median Weight (kg)  (IQR) | 57.0  (49.5 – 81.0) | 75.5  (62.4 – 92.5) | 67.5  (54.5 – 87.7) | **0.02** |
| Median Height (cm)  (IQR) | 159.0  (154.0 – 163.5) | 162.5  (159.0 – 168.0) | 161.5  (155.0 – 166.0) | **0.03** |
| Geographic Region, n (%) |  |  |  | 0.12 |
| North America | 5 (20.8) | 12 (40.0) | 17 (31.5) |  |
| South and Latin America | 15 (62.5) | 10 (33.3) | 25 (46.3) |  |
| Asia-Pacific | 4 (16.7) | 6 (20.0) | 10 (18.5) |  |
| Europe | 0 (0) | 2 (6.7) | 2 (3.7) |  |
| Median Time Since Diagnosis (months), (IQR) | 2.6  (0.12 – 8.8) | 11.3  (3.8 – 39.1) | 7.0  (1.9 – 16.1) | **0.01** |
| Background PAH Therapy, n (%) |  |  |  | 0.69 |
| PDE5-I or sGC Stimulator | 20 (83.3) | 27 (90.0) | 47 (87.0) |  |
| ERA Alone | 4 (16.7) | 3 (10.0) | 7 (13.0) |  |
| Median Time on Background PAH Therapy, (months), (IQR) | 5.8  (2.8 – 9.6) | 5.8  (3.0 – 12.4) | 5.8  (2.9 – 10.2) | 0.67 |
|  |  |  |  |  |

*p-values were calculated using Wilcoxon rank sum test for continuous variables and Fisher’s exact test for categorical variables. Abbreviations: ERA, endothelin receptor antagonist; PAH, pulmonary arterial hypertension; PDE5-I, phosphodiesterase type 5 inhibitor; sGC, soluble guanylate cyclase

**Supplemental Table E2. Summary of Hemodynamic Parameters-Raw Values**

|  | **Placebo** | | | | **Oral Treprostinil** | | | |  |
| --- | --- | --- | --- | --- | --- | --- | --- | --- | --- |
|  | **n** | **Baseline** | **Week 24** | **Change**  **(95% CI)** | **n** | **Baseline** | **Week 24** | **Change**  **(95% CI)** | **p-value^*^** |
| **PAC (mL/mmHg**) | 24 | 1.8 | 1.8 | -0.04  (-0.4 – 0.4) | 30 | 1.8 | 2.6 | 0.8  (0.4 – 1.1) | **0.004** |
| **CO (L/min)** | 24 | 4.8 | 4.5 | -0.4  (-0.9 – 0.2) | 30 | 5.3 | 5.7 | 0.5  (0.0 – 1.0) | **0.02** |
| **CI (L/min/m^2^)** | 22 | 3.0 | 2.7 | -0.3  (-0.6 – 0.0) | 26 | 3.1 | 3.3 | 0.2  (-0.1 – 0.5) | **0.03** |
| **PVR (dynes*sec/cm^5^)** | 24 | 781 | 772 | -1.5  (-106 – 103) | 30 | 720 | 586 | -140  (-234 – -46.4) | **0.05** |
| **SVR (dynes*sec/cm^5^)** | 24 | 1474 | 1562 | 92.5  (-59.1 – 244) | 29 | 1457 | 1285 | -175  (-313 – -37.0) | **0.01** |
| **PAWPm (mmHg)** | 26 | 9.2 | 8.6 | -0.8  (-2.5 – 0.8) | 33 | 10.0 | 11.0 | 1.18  (-0.3 – 2.65) | 0.07 |
| **PAPm (mmHg)** | 27 | 49.4 | 47.0 | -2.4  (-6.7 – 1.9) | 34 | 49.2 | 46.0 | -3.1  (-7.0 – 0.7) | 0.79 |
| **SAPm (mmHg)** | 27 | 86.7 | 86.9 | -0.3  (-5.2 – 4.6) | 33 | 90.2 | 90.5 | 0.7  (-3.7 – 5.2) | 0.75 |
| **RAPm (mmHg)** | 24 | 8.8 | 7.8 | -0.8  (-2.7 – 1.1) | 33 | 7.8 | 8.2 | 0.4  (-1.3 – 2.0) | 0.37 |
| **SvO2 (%)** | 21 | 65.9 | 69.7 | 3.4  (0.1 – 6.7) | 26 | 67.3 | 67.9 | 0.9  (-2.0 – 3.8) | 0.26 |
| **SaO2 (%)** | 26 | 95.8 | 95.5 | -0.2  (-1.5 – 1.1) | 32 | 95.0 | 94.8 | -0.3  (-1.5 – 0.8) | 0.89 |
| **NT-proBNP (ng/mL)** | 27 | 662 | 865 | 194  (-110 – 499) | 33 | 733 | 583 | -142  (-418 – 133) | 0.11 |

Hemodynamic parameters are expressed as means. Only subjects with both baseline and Week 24 hemodynamic measures were included. *p-value, change in the mean, and its associated 95% CI are obtained from the analysis of covariance with change from baseline in raw data for each hemodynamic parameter (e.g. NT-proBNP) as the dependent variable, treatment as fixed effect, and raw baseline hemodynamic parameter (e.g. NT-proBNP) as a covariate. Abbreviations: CO, cardiac output; CI, cardiac index; PVR, pulmonary vascular resistance; SVR, systemic vascular resistance; PAWPm, pulmonary artery wedge pressure mean; PAPm, pulmonary artery pressure mean; SAPm, systemic arterial pressure mean; RAPm, right atrial pressure mean; SaO2, arterial oxygen saturation; SvO2, mixed venous oxygen saturation
